# Physical Characterization of Multifiber Polyvinylidene Fluoride with the Addition of Hexafluoropropylene and/or Graphene Oxide

**DOI:** 10.3390/polym17223037

**Published:** 2025-11-16

**Authors:** Lorenzo Torrisi, Angela Malara, Antonio Fotia, Chiara Nunnari, Patrizia Frontera, Alfio Torrisi, Gabriele Salvato, Letteria Silipigni, Mariapompea Cutroneo

**Affiliations:** 1 Dipartimento MIFT, Università di Messina, Viale F.S. d’Alcontres 31, 98166 Messina, Italymcutroneo@unime.it (M.C.); 2 Department of Civil, Energetic, Environment and Material Engineering, Mediterranean University of Reggio Calabria, 89124 Reggio Calabria, Italy; angela.malara@unirc.it (A.M.);; 3 CNR-ITAE, Istituto di Tecnologie Avanzate per l’Energia, 98125 Messina, Italy; 4 Dipartimento di Medicina e Chirurgia, Università di Enna “Kore”, 94100 Enna, Italy; alfio.torrisi@unikore.it; 5 CNR-IPCS, Viale F.S. d’Alcontres 37, 98166 Messina, Italy; gabriele.salvato@cnr.it

**Keywords:** PVDF, HFP, GO, nanofiber, electrospinning, polymer properties

## Abstract

Multifiber polyvinylidene fluoride (PVDF), a thermoplastic polymer, was produced as a one-dimensional nanostructure via the electrospinning technique. Due to the peculiar properties attributed to the nanoscale fiber dimension, PVDF material, as pure, and with the addition of hexafluoropropylene (HFP) and/or graphene oxide (GO), was thoroughly characterized in terms of morphology, density, optical and electrical properties, surface wettability, mechanical resistance, and other physical characteristics. PVDF, with a multifiber surface, with or without the addition of other elements, has been demonstrated to have a strong capacity to absorb high concentrations of gases, water, nanoparticles, and other substances. The material’s dielectric behavior and soft and shock-absorbing polymer properties make it ideal for biocompatible applications, which will be showcased and discussed in this work. A detailed comparison was made between bulk PVDF, multifiber PVDF, and PVDF containing HFP and/or GO, highlighting the changes in polymer properties.

## 1. Introduction

Polyvinylidene fluoride (PVDF) is a semi-crystalline thermoplastic fluoropolymer that is readily melt-processible, can be fabricated by injection and compression molding, can be used up to about 150 °C, combines high mechanical strength with good processability, and has strong piezoelectric properties.

**Figure 1:**
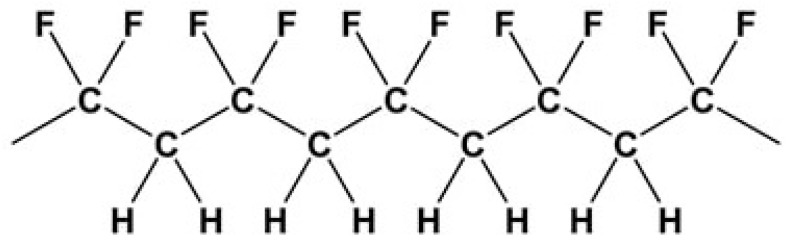
Molecular structure of polyvinylidene fluoride (PVDF).

The market for polymers finds PVDF to be an interesting material due to its versatility in uses. In high-tech applications, it is widely utilized. These include mainly chemical process equipment and electrical and electronics applications. The molecular structure of PVDF is constituted by chains with alternating CH_2_ and CF_2_ groups, as described in Figure 1:

The PVDF melting point is 178 °C, the density 1.78 g/cm^3^, the tensile strength and modulus at 23 °C are about 50 MPa, and 1700 MPa, respectively, the coefficient of thermal expansion is about 10^−4^, the dielectric constant at 1 kHz is about 10, the dissipation factor at 1 kHz about 0.018, its resistivity is about 10^16^ Ω cm [1]. PVDF has high thermal stability, chemical resistance, biocompatibility, and processability. It is used in the biomedical field to realize biomembranes. PVDF is a hydrophobic polymer; its water absorption over 24 h is very low, about 0.04 wt%.

PVDF is a semicrystalline polymer containing one or more crystalline phases, known as α, β, γ, δ, dispersed in the amorphous regions [2]. The PVDF structure with chemical composition -C_2_H_2_F_2_- has a piezoelectric behavior because of the dipoles formed by the positive hydrogen atoms and the negative fluorine. Nanometric multifibers are densely packed on the surface of a PVDF foil produced using the electrospinning deposition process. The surface is able to absorb water, gases, nanoparticles, and micromaterials with great efficiency.

Electrospinning is a simple method for producing nanofibers or nanowires, resulting in large surface areas and highly porous structures with diameters ranging from nanometers to micrometers. This deposition process uses an electrostatic field, applied between the metallic needle emitting the molecular spray and the substrate on which the film will be deposited, which permits control over the fiber dimensions and morphology [3]. Moreover, the incorporation of fillers during electrospinning permits the realization of more complex structures with peculiar properties, such as those obtained by inserting GO nanoparticles (NPs) in polystyrene and polymethylmethacrylate [4,5].

Graphene oxide is a compound of carbon, oxygen, and hydrogen in variable ratios, obtained by treating graphite with strong oxidizers and acids. Graphene oxide can be reduced by ion irradiation [6], laser irradiation [7], and other approaches to obtain reduced graphene oxide (rGO). Generally, the C/O ratio ranges between 2 and 3, and the oxygen content is significant. GO’s properties become more like graphene’s when its oxygen content is reduced, leading to an increase in density and electrical conductivity. GO has a low density of about 1.5 g/cm^3^ and is an insulating material [8].

Hexafluoropropylene (HFP) is the fluoroalkene with the formula CF_3_CF = CF_2_, i.e., with a chemical compositional -C_3_F_6_-, with an F/C atomic composition 2/1, equal to that of polytetrafluoroethylene. Its production is possible through the pyrolysis of tetrafluoroethylene. It is insulating, hydrophobic, and has properties similar to those of CF_2_ [9].

Literature reports that the PVDF-HFP copolymer contains amorphous domains capable of trapping a large amount of liquid electrolytes, and its crystalline phase acts as a mechanical support [10]. The chemical and physical properties of the multifiber polymer were modified by embedding both GO and HFP nanoparticles into PVDF at a high concentration (from 10 to 50 wt%). In particular, the GO embedment should enhance the composite polymer’s capacity to absorb gases and liquids and to change the optical properties, making the polymer dark and absorbent of visible light, as reported in the literature [11]. While the HFP should enhance the hydrophobic capacity of the polymer fibers, enhancing their mechanical and thermal resistance and durability [12]. From literature data and for these motifs, the proposed copolymer represents an innovative material with respect to the previously PVDF copolymer, which has demonstrated insufficient resistance and damage in hazardous chemical environments [13]. Literature reports that the chemical and physical properties of PVDF can be strongly altered by the presence of copolymers, doping, and treatments [14].

With the present investigations, we are trying to create an innovative PVDF composite that can overcome the difficulties of the basic polymer in filtering liquids and gases and in absorbing certain molecular species, to address open problems on desalination and air filtration in the presence of toxic gases [15]. For these motifs, the use of nanoparticles embedded into the polymer is investigated using a high weight concentration of about 10% and not using low concentrations, although we know that such contents can alter the mechanical properties of the starting polymer. In the ambit of polymer synthesis with innovative techniques using ion beams, lasers, UV and X-rays, electron beams, and electro-spinning, the insertion of other molecules in its structure significantly changes the physical and chemical properties [16,17,18,19]. This research investigates the physical properties of electrospinning multifiber PVDF without and with the insertion of GO and HFP, concerning those of the pristine PVDF bulk, to put in evidence the advantages and peculiarities of the obtained multifiber compounds.

## 2. Materials and Methods

Polyvinylidene fluoride (PVDF) and polyvinylidene fluoride with the addition of hexafluoropropylene (PVDF-HFP), dimethylformamide (DMF), acetone, and graphene oxide (GO) powder (average number of layers, 15–20) were used for the production of fibers or wires. All reagents, Poly(vinylidene fluoride) (PVDF, Mw 530,000), Poly(vinylidene fluoride-co-hexafluoropropylene) (PVDF-HFP, Mw 455,000) were purchased from Sigma Aldrich (Burlington, Massachusetts, United States) and they were used without any further modification.

### 2.1. Sample Preparation

The production of electrospun microfibers started with the preparation of the polymeric solutions, according to the following procedure: for PVDF microfibers, the pellets were dissolved in a mixture of DMF and acetone and then stirred at 250 RPM for 2 h at 50 °C. Instead, to obtain the PVDF-GO, the pellets were first dissolved in DMF and acetone, stirred at 250 RPM for 2 h at 50 °C, and finally, GO was dissolved in the solution and stirred at 250 RPM for 24 h. Similarly, the same procedure was followed in the case of both samples, PVDF-HFP and PVDF-HFP-GO.

For the production of electrospun material processes, polymeric solutions were introduced into a 10 mL glass syringe equipped with a 1 mm metal needle. A pump was utilized to regulate the flow rate at approximately 0.020 mL/min. The electrospinning processes were conducted at 25 °C with a relative humidity of 40%. Table 1 summarizes the electrospinning parameters used to produce each sample.

### 2.2. Techniques of Analysis

The Attenuated Total Reflectance (ATR) coupled to the Fourier Transform Infrared (FTIR) spectroscopy was employed to characterize the transmittance and absorbance of the investigated polymers. A Jasco (Tokyo, Japan) FT/IR 4600 spectrometer was employed in the wavenumber range 400–4500 cm^−1^ with a resolution of 4 cm^−1^. The number of scans used for ATR-TFIR measurements is 5, and the tip used is diamond.

UV-Visible optical spectroscopy was performed in the wavelength region 250–850 nm by using the Jasco (Tokyo, Japan) V-750 double-beam spectrophotometer for high-resolution measurements, with a spectral bandwidth that can be set as narrow as 0.1 nm.

The morphological characterization of electrospun microfibers was performed using scanning electron microscopy (SEM) (Phenom ProX, Thermo Fisher Scientific, Waltham, MA, USA). For SEM analysis, samples were dried in an oven at 50 °C for 24 h, then mounted on aluminum stubs using carbon adhesive tape. To improve image acquisition and minimize charging effects, a thin graphite coating was applied to the surface.

The optical microscopy was used for preliminary observation of the prepared polymers and measurements of polymer surface wetting. Based on the sessile drop technique, the wetting angle of distilled water was measured at room temperature, 1 atm pressure, and 50% humidity, using drops of 2 μL volume deposited with a micro-syringe on the polymer surface and PC controlled online through the optical microscope at 8× magnification. Each measurement of the contact angle was ensured by flat and accurate polishing of the surfaces.

SEM images were acquired using a Phenom Pro X scanning electron microscope and analyzed with the corresponding Phenom Pro Suite software (v. 5.8.1), specifically the Fibermetric and Porosimetric modules, to give information on the fiber size and porosity.

XRD spectra were collected by a Panalytical Empyrean ((Malvern Panalytical, Almelo, The Netherlands) S-2 diffractometer, using Cu Ka radiation (1.54056 Å) at 40 kV and 40 mA. The patterns were recorded in step scan mode from 10 to 60° 2θ angles in steps of 0.02° and a count time of 5 s per step.

The electrical characterization of the investigated polymers was performed by analyzing the frequency dependence of the real part ε1 of permittivity, the imaginary part ε2 of permittivity, and the loss tangent tan δ in the (10^3^ ÷ 10^6^) Hz range at room temperature using an Agilent 4284A precision LCR meter [20]. The samples were sandwiched between two symmetric stainless-steel electrodes of the Keysight 16451B dielectric material test fixture, and a 2 V peak-to-peak voltage was applied. The guarded electrode had an area of about 19.64 mm^2^.

Electrospun fibers were tested for tensile strength to investigate their mechanical behavior. For the tensile strength test, a Zwicki (ZwickRoell, Ulm, Germany) 2.5 kN machine coupled with a load cell of 200 N was used; the gauge length was fixed at 30 mm, and the tensile speed was set at 0.5 mm/min. To obtain an average value, three fiber samples were tested for each polymer. The specimens used for tensile testing were dog-bone shaped, in accordance with the relevant standard, and had a gauge length of 50 mm; they all had the same width of 8 mm. Results were normalized by sample thickness.

## 3. Results and Discussion

The first investigation concerns the SEM observations of the polymer morphology without and with the GO and HFP components.

Many samples have been investigated using SEM, optical spectroscopy, besides other techniques, and our analysis concerned:

(1) PVDF foils, 100 microns thickness, investigated as bulk, without and with 10 wt% GO, and as electrospun multifiber with and without 10 wt% GO.
(2) PVDF-HFP foils, 100 microns thickness, investigated also as bulk, without and with 10 wt% GO, and as electrospun multifiber with and without 10 wt% GO.

**Figure 2:**
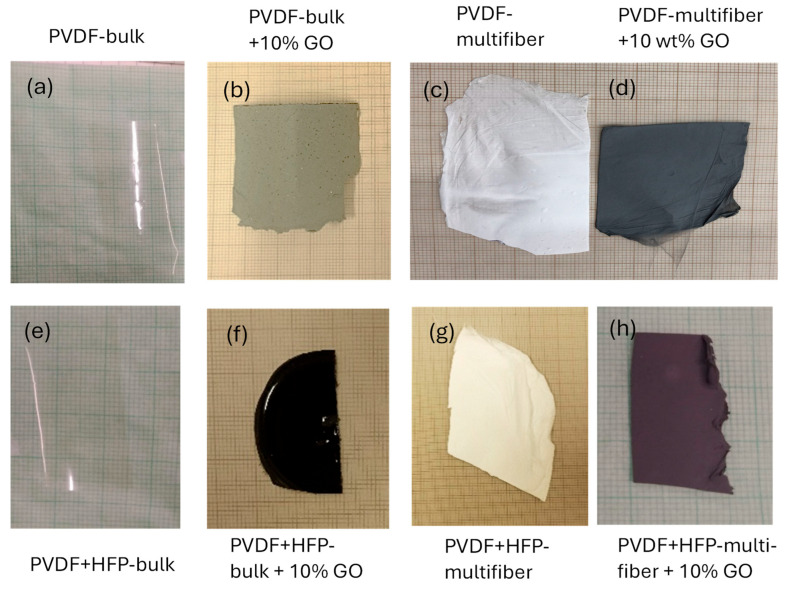
Photos of the eight types of different investigated polymers: PDVF bulk (**a**), PVDF bulk + 10 wt% GO (**b**), PVDF multifiber (**c**), PVDF multifiber + 10 wt% GO (**d**), PVDF + HFP-bulk (**e**), PVDF + HFP-bulk + 10% GO (**f**), PVDF + HFP-multifiber (**g**), and PVDF + HFP-multifiber + 10% GO (**h**).

Figure 2 reports the photos of the eight types of different polymer objects of this investigation: PDVF bulk (a), PVDF bulk + 10 wt% GO (b), PVDF multifiber (c), PVDF multifiber + 10 wt% GO (d), PVDF + HFP-bulk (e), PVDF + HFP-bulk + 10% GO (f), PVDF + HFP-multifiber (g), and PVDF + HFP-multifiber + 10% GO (h). The PVDF bulk foils, whether they have HFP or not, are transparent. The polymers containing GO are gray or black, the spun multifiber polymers are white if not contain GO or black if they contain GO nanoparticles.

**Figure 3:**
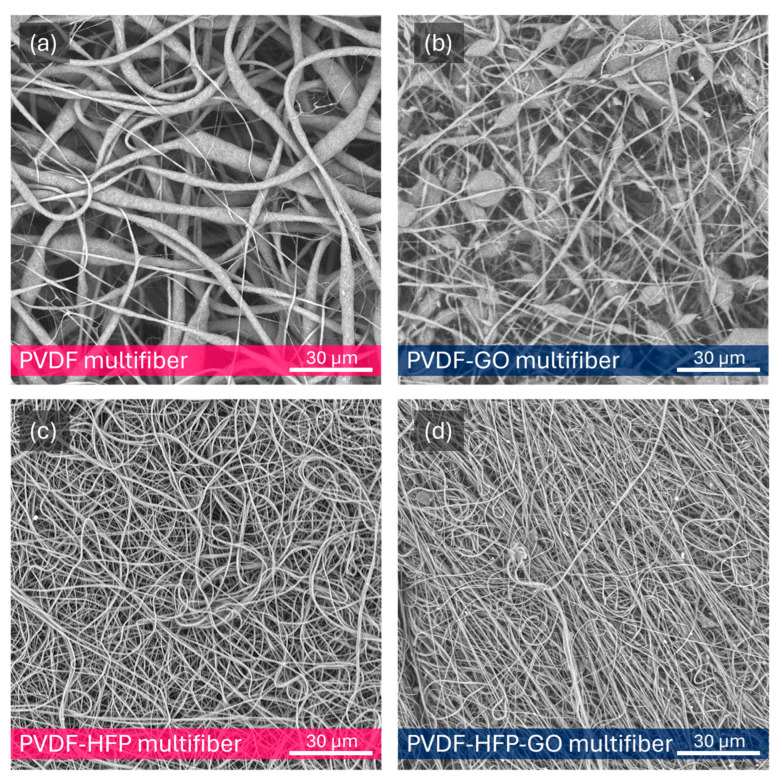
SEM image of multifiber pristine PVDF (**a**), PVDF with 10 wt% of GO NPs (**b**), PVDF-HFP (**c**), and PVDF-HFP with 10 wt% GO (**d**).

Electron microscopy, performed at 10 kV and generally at 2000×, has shown a high degree of micrometric diameter fiber density distributed in random directions with a length of the order of 0.1–1 mm. Figure 3a reports an SEM photo of the pristine pure multifiber PVDF fibers’ morphology at 2000× magnification, indicating an average fiber diameter of 3 μm. Figure 3b reports on the multifiber PVDF with 10 wt% GO nanoparticles. In this case, it is possible to observe a minor fiber diameter and the presence of many micrometric beads. Typically, solvent evaporation rate impacts fiber solidification (i.e., diameter, bead formation). Dimethylformamide (DMF) is a low-volatility solvent, affecting the features of the electrospinned PVDF. The average fiber diameter goes down to 1.2 mm.

Figure 3c reports the SEM image of the multifiber PVDF with 50 wt% HFP, indicating a minor fiber diameter, with an average value goes down to about 0.75 mm. Figure 3d is relative to the image of the PVDF containing both HFP and GO, at 50 wt% and 10 wt%, respectively, showing a further fiber diameter reduction; in fact, its average value goes down to 0.55 mm.

**Figure 4:**
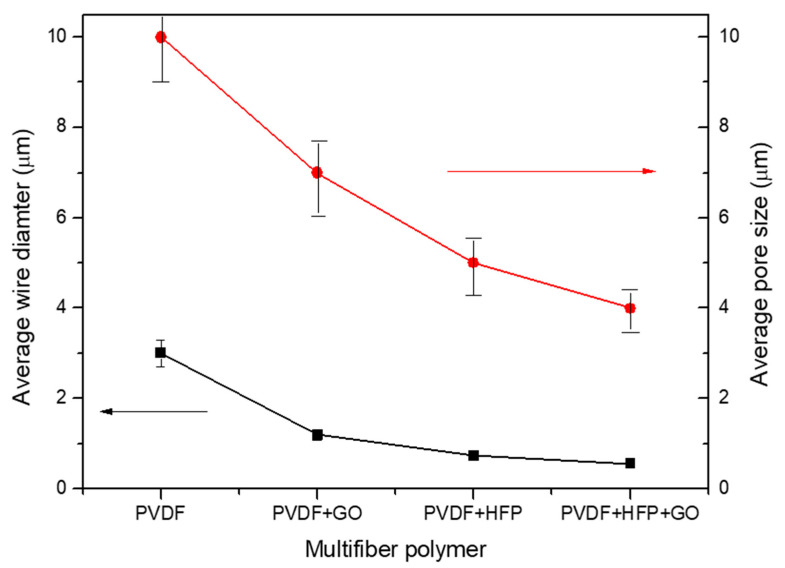
Average fiber diameter versus type of PVDF copolymer used.

Thus, a first result derives from the fibers’ diameter decreasing, from about 3 mm up to about 0.5 mm, with the insertion in the PVDF of GO, HFP, and HFP + GO, as evident from the plot of Figure 4, where the fiber diameter measurements have a bar error of about 10%. The average fiber diameter decrease produces a minor polymer porosity, a higher polymer density, and a multifiber density enhancement, changing the final properties of the sample. Figure 4 also reports the pore average size in the case of the four types of electrospun polymers. The pore size decreases with the fiber diameter, from about 10 mm at high fiber diameters up to about 4 mm at low fiber diameters.

**Figure 5:**
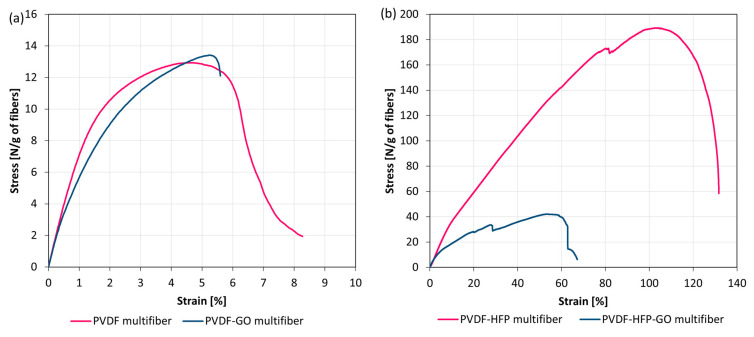
Stress–strain curves for multifiber PVDF with and without GO (**a**) and PVDF-HFP with and without GO (**b**).

The mechanical properties of the multifibers were obtained by applying a tensile strength test, measuring the stress as a function of the strain. Figure 5 reports the obtained results for PVDF multifiber with and without GO (a) and those for PVDF-HFP multifiber with and without GO (b). The mechanical properties of the multifiber systems revealed substantial differences depending on the polymer matrix and the incorporation of GO.

The presence of HFP and GO generally reduces the fiber diameter, especially if both substances are embedded. This effect is due to macroscopic and microscopic effects. The macroscopic ones concern the overall changes in solution viscosity and thermal conductivity, because a high concentration of nanoparticles is employed, and it is possible that nanoparticle agglomeration is produced, influencing the electrospinning deposition as a minor final fiber thickness. The microscopic ones concern the overall change in electrical conductivity and charging effects due to electron trapping at the PVDF-GO and PVDF-HFP interfaces, which, even if only slightly, modify the electrical conditions of the electrospinning deposition process, giving rise to a minor fiber diameter. Such fiber reduction with the high concentration nanoparticle inclusions is in agreement with the literature data [21,22].

The pristine PVDF multifiber displayed moderate tensile strength (≈13 N/g of fibers) and a relatively low strain at break (≈6.5%), indicating a material with limited ductility and only modest load-bearing capacity. When GO was introduced into the system, the PVDF-GO multifiber exhibited a slight increase in tensile strength (≈13.5 N/g), while maintaining a similar strain at break (≈6.0%). This modest improvement suggests that GO interacted favorably with the PVDF matrix, possibly acting as a stress-transfer medium without negatively impacting deformability. Although the absolute gain was small, it points to a potential reinforcing effect of GO in pure PVDF systems. In contrast, the PVDF-HFP multifiber demonstrated outstanding mechanical performance, combining a very high tensile strength (≈190 N/g) with exceptional elongation at break (≈130%). This remarkable balance of strength and ductility indicates a tough material capable of withstanding both high stresses and large deformations. However, the incorporation of GO into the copolymer drastically altered this behavior. The PVDF-HFP-GO multifiber exhibited a sharp reduction in mechanical properties, with maximum stress dropping to ≈40 N/g and strain at break limited to ≈60%. The stress–strain curve also revealed an unstable response with earlier failure, consistent with a brittle fracture. These findings suggest that GO was not well integrated into the PVDF-HFP matrix, likely due to incompatibility or poor dispersion, resulting in the formation of structural defects and localized stress concentrators.

Overall, the comparative analysis highlights two distinct scenarios: in PVDF, GO acted as a mild reinforcing agent, whereas in PVDF-HFP, it compromised the excellent intrinsic toughness of the copolymer. This dual behavior emphasizes the critical role of matrix composition in determining the reinforcing efficiency of nanofillers. It also underscores the importance of optimizing filler–matrix compatibility and dispersion to achieve the desired mechanical performance in polymer nanocomposites.

**Figure 6:**
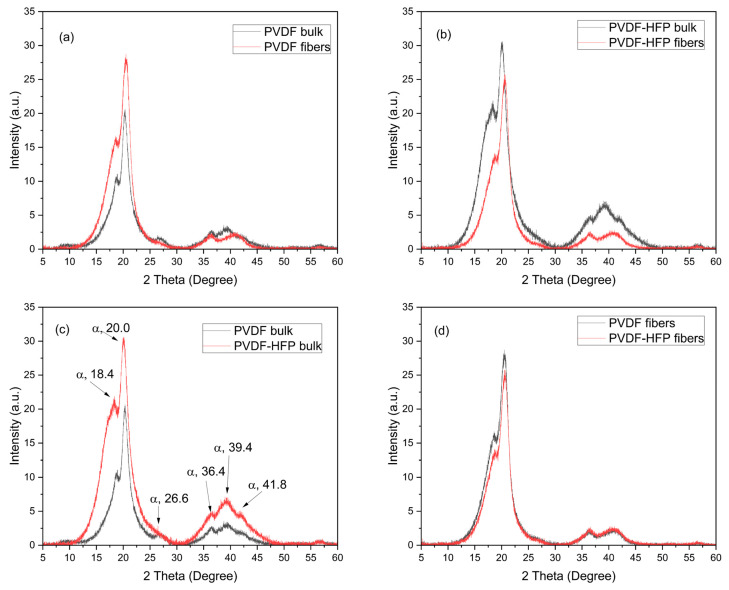
XRD spectra relative to the comparison PVDF bulk and fibers (**a**), PVDF-HFP bulk and fibers (**b**), PVDF and PVDF-HFP bulk (**c**), and PVDF and PVDF-HFP fibers (**d**).

XRD analysis evinces the crystallinity of the investigated polymer and puts in evidence the differences between the bulk and the multifiber polymer. Moreover, it shows the difference in PVDF when HFP is added at 50% modifying the matrix composition. The main results of the XRD analysis are reported in the spectra comparison shown in Figure 6. The spectra indicate that PVDF is a semicrystalline polymer exhibiting different crystalline forms, especially the most common α, β, and γ phases. The XRD analyses indicate that the XRD patterns of the PVDF foils exhibited two intense diffraction peaks at 18.4 and 20.0° and a low peak at 26.6°, as indicated in the spectrum of Figure 6c, corresponding to 020, 110, and 021 reflections of the monoclinic α-phase crystal, respectively [23,24]. Also, the three weak peaks at 36.4, 39.1, and 41.8°, indicated in Figure 6c, correspond to 200, 002, and 111 reflections of the monoclinic α-phase crystal, respectively [24]. Thus, well evident is the α-phase of the polymer, while the phases β and γ are less evident due to weaker diffraction peaks.

Figure 6a indicates that the crystallinity is higher in the multifiber case concerning the bulk PVDF foil, probably due to the most exposed surface in the case of the multifiber sample. Figure 6b indicates that the PVDF-HFP polymer bulk has a crystallinity higher than the multifiber version. The insertion of HFP in PVDF and the electrospinning process does not significantly alter the angular position of the XRD diffraction peaks. The narrowing of the diffraction peaks significantly reduces the intensity, indicating that, even at high concentration, its diffraction appears like that of an amorphous material, without typical diffraction peaks. Figure 6c shows that the crystallinity in PVDF bulk is much less than in the PVDF-HFP bulk polymer, which maintains high semicrystallinity. Finally, Figure 6d indicates that the PVDF and the PVDF-HFP multifiber polymers have a very similar crystallinity, confirming the absence of crystallinity in the added HFP copolymer.

**Figure 7:**
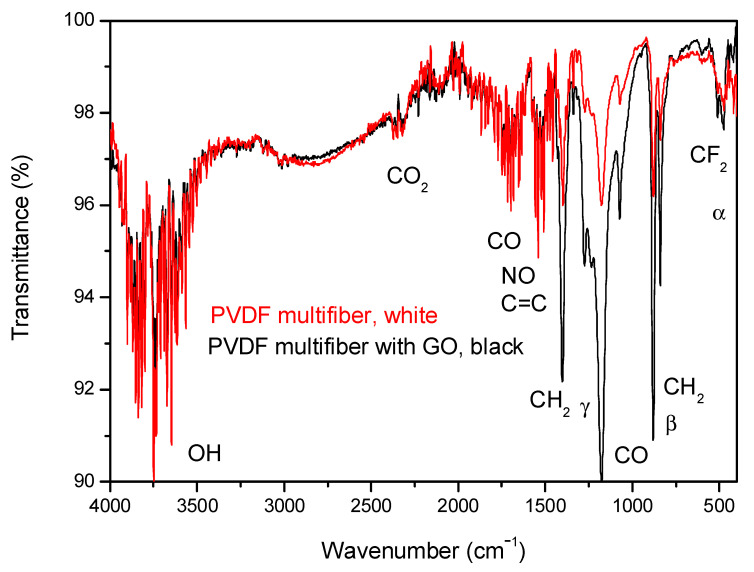
ATR-FTIR spectra comparison between PVDF bulk and PVDF multifiber with and without GO nanoparticles.

A further analysis of the characterization of the four multifiber polymers, including the PVDF-based one, has been performed using ATR-FTIR spectroscopy. Figure 7 reports the FTIR transmittance spectra comparison between the investigated multifiber polymers: PVDF multifiber, with a white color, and PVDF multifiber with 10 wt% GO, with a black color. The average transmittance is about 98% with a slight decrease for the black PVDF due to its inclusion of GO absorbent gas, water vapor, and CO groups.

Such spectra are complex because they show many characteristic absorption peaks due to the presence of the different crystallographic phases of the polymer. The characteristic bands of α phase are around at 410, 489, 532, 614, 763, 795, 854, 975, 1149, 1209, 1383 and 1423 cm^−1^, whereas characteristic bands of the β and γ phases are around at 445, 473, 1275 and 1431 cm^−1^ and around at 431, 482, 811, 1234 and 1429 cm^−1^, respectively, according to the literature [24,25,26]. The α, β crystalline phases are the main components, while the γ phase is negligible.

The strong peak for the CO group at 1178 cm^−1^ and that of NO at 1538 cm^−1^ indicate high oxygen and air (nitrogen) absorption of the multifiber species without GO due to its high porosity generated between the fibers. It decreases in the case of PVDF with GO, which has a minor porosity, but remains as the typical functional groups of oxygen present in this material [27].

The spectra have similar IR absorption peaks, decreasing the average transmittance due to the significant vibrational states of the groups of OH, CO_2_, CO, NO, CH_2_, and CF_2_. The C = C contribution comes from the GO addition. The PVDF containing GO has a lower transmittance in the wavenumber region 1500–500 cm^−1^, due to the high concentration of GO nanoparticles and to the high content of functional groups of oxygen.

**Figure 8:**
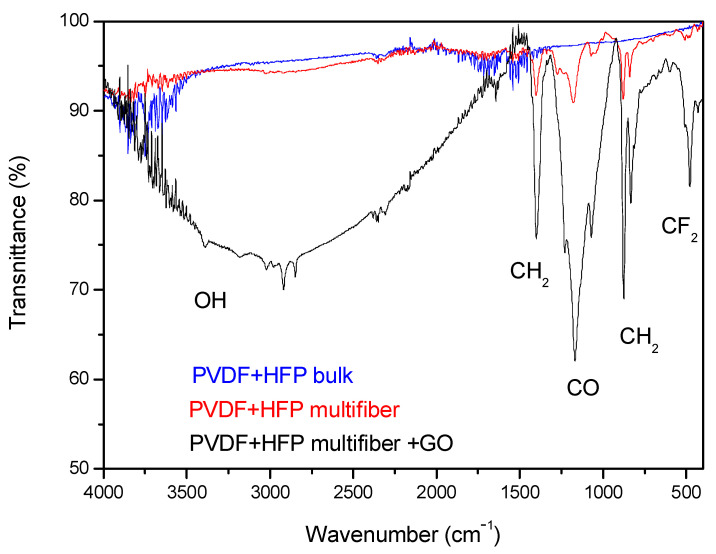
ATR-FTIR spectra comparison between PVDF-HFP bulk and PVDF-HFP multifiber with and without GO nanoparticles.

Figure 8 reports the FTIR transmittance spectra comparison between the investigated multifiber polymers: PVDF-HFP bulk, which is transparent to visible light, PVDF-HFP multifiber, with a white color, and PVDF-HFP multifiber with 10 wt% GO, with a black color. The average transmittance is about 95% in the case of PVDF-HFP bulk and multifiber, but decreases to about 90% or less in the case of PVDF-HFP multifiber with GO, due to different molecular groups being absorbed by both the multifiber and the GO. In fact, GO contains at high concentration different functional groups of oxygen, such as carboxylic, carbonylic, hydroxyl, epoxy, and water, which have characteristic absorption peaks in the IR region. In the case of black polymer, the GO, although at a concentration of 10 wt%, can absorb much more water, from which the large OH band around 3000 cm^−1^ wavenumber occurs, and CF_2_ groups, in addition to the usual oxygen functional groups, than in the case of PVDF + HFP multifiber and bulk version.

A further characterization of the investigated polymers was performed using the sessile drop method of wetting their surface with water at room temperature (20 °C), atmospheric pressure (1 atm), and 50% humidity.

**Figure 9:**
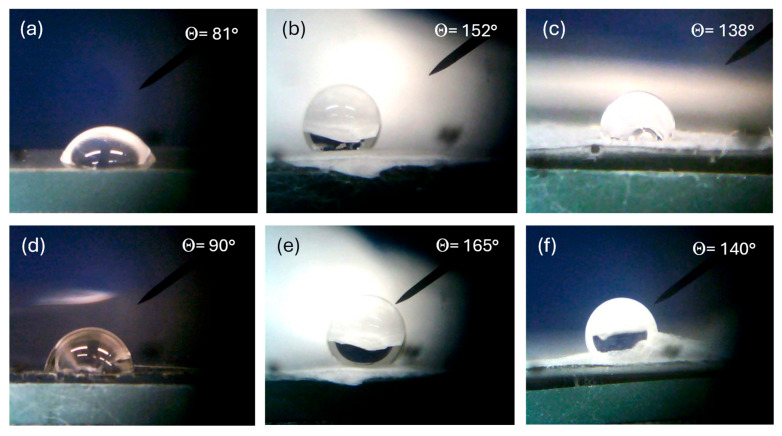
Contact angle for PVDF bulk (**a**), PVDF multifiber (**b**), PVDF multifiber with GO (**c**), PVDF-HFP bulk (**d**), PVDF-HFP multifiber (**e**), and PVDF-HFP with GO (**f**).

All such polymers are hydrophobic, but their degree of hydrophobicity depends on the composition and morphology of the surface. Figure 9 reports a lot of optical microscope photos of a 2 mL distilled water drop deposited on the surface of different types of polymers. In particular, Figure 9a shows the contact angle with the bulk PVDF surface of 81°, Figure 9b that of PVDF multifiber of 152°, and Figure 9c that of PVDF multifiber with 10 wt% GO nanoparticles, of 138°. Thus, for this polymer, the hydrophobicity increases significantly from the bulk morphology to the phase of electrospun multifiber and then decreases due to the insertion of the GO nanoparticles. Similar results, but with a higher hydrophobicity, have been obtained for the PVDF-HFP polymer, showing a contact angle of 90° for the bulk version, 165° in the case of PVDF + HFP multifiber, and 140° for the PVDF + HFP + 10 wt% GO.

**Figure 10:**
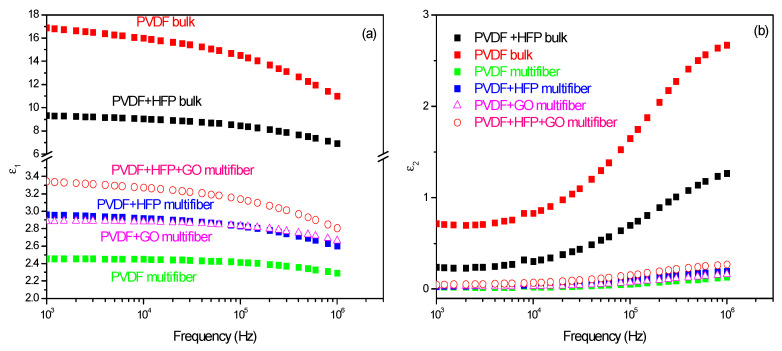
Dielectric constant real (**a**) and imaginary (**b**) as a function of the frequency and of the polymer type.

The dielectric constant measurements performed on the various investigated polymers have been compared, and the results, plotted as a function of frequency and type of polymer, are summarized in Figure 10 [28]. Figure 10a reports the dielectric real constant e1 as a function of the frequency and of the type of analyzed polymer. The bulk matter has a higher dielectric constant (of the order of 10–17) than the multifiber (of the order of 2.4–3.4), as expected due to the presence of air and water vapor between the fibers. The insertion of HFP and GO in PVDF enhances the dielectric constant. Figure 10b reports the dielectric imaginary constant e2 as a function of the frequency and of the type of analyzed polymer. Also, in this case, the bulk matter has a higher dielectric constant than the multifiber, and the insertion of HFP and GO in PVDF enhances the dielectric imaginary constant, maintaining the order observed for the real component.

Furthermore, measurements of the ratio of the loss current to the charging current, i.e., the loss tangent tan d, and of the polymer’s weak electrical conductivity s have been acquired as a function of the frequency and of the type of investigated polymer.

**Figure 11:**
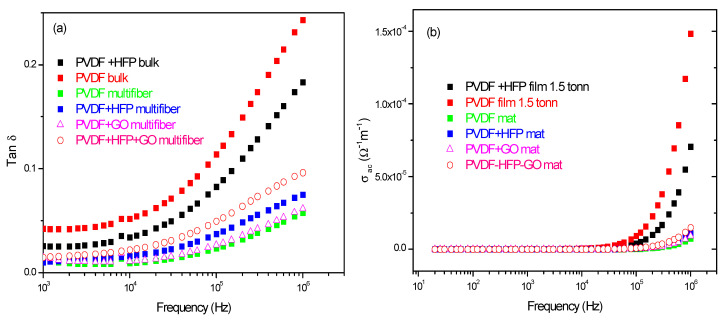
Tan d (**a**) and electrical conductivity (**b**) vs. frequency and polymer type.

Figure 11a reports the comparison between the measurements of tan d vs. frequency and polymer type. The tan δ has higher values for the PVDF and PVDF + HFP bulk foils and minor values for the multiwfiber polymers, of which the higher value is obtained for the PVDF + HFP + GO, maintaining the previous reported order. From such data, it is possible to evaluate the weak electrical conductivity of the different polymers vs. frequency, as reported in Figure 11b. For all polymers, the electrical conductivity is practically zero from low frequency up to about 1 MHz; only for higher frequencies it shows some increment at values of the order of 10^−4^/Ωm.

Some words about the possible application of the investigated polymers. PVDF with and without HFP and GO may find interesting applications in different fields. The possibility to use them as multifibers with different densities, diameters, and lengths allows them to be used in creating membranes and filters that trap various liquid, solid, and gaseous substances between the fibers [29]. The absorption of water and higher-viscosity liquids is well known, while the absorption of micro- and nanoparticles is also possible thanks to the high porosity of multifiber polymers. Gas absorption is possible, especially with the inclusion of GO nanoparticles that trap various types of gases, such as oxygen, and retain various oxygen functional groups. Thus, polymers can be employed to filter gases for toxic species, to filter water for desalination, and to accumulate certain species of micro- or nanoparticles during industrial processes, acting as filters for polluting material.

Further applications can be found in the biomaterials, microelectronics, chemical treatments, optical devices, sensors, and others, as reported in the literature [30,31,32,33].

## 4. Conclusions

Innovative polymers for their production as a multifiber have been compared to their bulk version and modified by inserting another copolymer and/or carbon nanoparticles as graphene oxide. The results of these electrospinning processes produce polymers with peculiar properties, especially for filtering and absorbing high quantities of liquids, gases, and micro and nanoparticles.

The investigated polymers are based on the properties of PVDF and were electron-spun using the addition of GO and HFP copolymer separately, or together. The obtained copolymers have been studied from the point of view of their nano and micrometric morphology, diffraction patterns for the semi-crystalline nature, IR transmission spectroscopy to identify the main vibrational molecular groups essentially based on C, O and H, to understand their elasticity from stress–strain response, to measure their wetting ability on different surfaces, and to evaluate their dielectric constant for the dielectric behavior as a function of frequency. Such additions to PVDF polymer chains, in fact, modify the chemical and physical properties of the copolymer, making it more suitable for certain applications.

The most important innovation is due to the electrospinning process developed using peculiar characteristics and to the ability to add to PVDF other polymeric species, such as the HFP, or to add the insulator nanoparticles of GO, and adding together HFP and GO nanoparticles. These additions modify the fiber densities, the density and porosity of the fibrous polymer, the mechanical and elastic response, the semicrystalline structures determined by their lattice, the composition of the main molecular groups related to PVDF, the dielectric properties, and the wettability of their surfaces, which are always highly hydrophobic.

Indeed, the simplicity and scalability of the proposed technique would make it easy to use in any field, flexible in material composition, and not expensive for commercialization purposes. They find applicability to membranes, air filtration and protection, water filtration and desalination, retention of fine dust and toxic gases, sensors, tissue engineering, medical prostheses, flexible electronic support, and other applications. Next, our research will concern the use of such innovative multifibers to realize desalination microfilters and selective gas filters. The first is for human consumption or seawater irrigation, and the second is for absorbing specific toxic gases during industrial accidents or unwanted air contamination.

## Figures and Tables

**Table 1:** Summary of the electrospinning parameters used to produce each sample.

Sample Code	Weight Ratio Polymer/Solvent	Weight Ratio Additive/Polymer	Voltage (kV)	Flow Rate (μL/min)	Needle to Collector Distance (cm)
PVDF	16.0/84.0	-	12.5	20.0	15.0
PVDF-HFP	16.0/84.0	-	12.0	20.0	15.0
PVDF-GO	16.0/84.0	10.0/90.0	11.5	20.0	15.0
PVDF-HFP-GO	16.0/84.0	10.0/90.0	12.0	20.0	15.0

## Data Availability

The raw data supporting the conclusions of this article will be made available by the authors on request.
